# Editorial: Role and mechanism of regulated cell death in musculoskeletal development, homeostasis, and diseases

**DOI:** 10.3389/fcell.2025.1602778

**Published:** 2025-04-14

**Authors:** Sheng Chen, Xiaohao Wu, Chao Chen

**Affiliations:** ^1^ Department of Orthopaedics, Union Hospital, Tongji Medical College, Huazhong University of Science and Technology, Wuhan, China; ^2^ Division of Immunology and Rheumatology, Stanford University, Stanford, CA, United States

**Keywords:** regulated cell death, ferroptosis, cuproptosis, autophagy, osteoarthritis, intervertebral disc, rheumatoid arthritis, musculoskeletal disease

Cell death is an area of biomedical research that is rapidly evolving. Presently, cell death is classified into two main categories based on the involvement of specific molecular mechanisms: regulated cell death (RCD) and accidental cell death (ACD). ACD occurs when cells are subjected to extreme pressure, temperature, radiation, or chemical environments, resulting in an immediate and uncontrollable disruption of cellular structures. Since ACD lacks dedicated molecular machinery, it cannot be modulated through pharmacological or genetic interventions. In contrast to ACD, RCD is extensively involved in the regulation of cellular homeostasis through controlled processes that rely on specific molecular pathways. RCD, often referred to as programmed cell death under normal physiological conditions, plays a crucial role in tissue regeneration and development. However, under pathological conditions, RCD can often be triggered by harmful stimuli from the extracellular or intracellular environment when adaptive responses are insufficient. Given its crucial role in pathophysiological processes and the controllability of its molecular mechanisms, RCD has garnered significant attention in the field of cell death research, emerging as a highly popular and promising research direction. Since 1970s, numerous forms of RCD have been identified, such as ferroptosis, cuproptosis, apoptosis, autophagy-dependent cell death, lysosome-dependent cell death, mitochondrial permeability transition-driven necrosis, oxeiptosis, entotic cell death, necroptosis, NETotic cell death, alkaliptosis, parthanatos, pyroptosis, immunogenic cell death, and PANoptosis. Musculoskeletal disorders are the primary source of disability worldwide, resulting in immense human distress and substantial socioeconomic costs. The musculoskeletal system consists of bones, skeletal muscles, joints, and associated tissues. It performs many vital functions, such as enabling mechanical movement, supporting body weight, maintaining posture and body shape, storing minerals, functioning as hematopoiesis, and protecting internal organs. These functions depend on the good quality and adequate quantity of cells within the musculoskeletal system. But aging and microenvironmental changes can lead to increased cell death and diminished cellular function, contributing to the onset of musculoskeletal diseases. RCD is strongly linked to the onset and progression of musculoskeletal disorders, particularly those affecting the synovium, bone, cartilage, and intervertebral disc (IVD), including rheumatoid arthritis (RA), osteoarthritis (OA), and degeneration of the IVD (IVDD). In patients with RA, alterations in iron metabolism result in iron accumulation, which stimulates the generation of reactive oxygen species (ROS) through the Fenton reaction. The elevated ROS level subsequently accelerates lipid peroxidation and impairs cellular membranes, ultimately leading to ferroptosis. The cellular damage induced by ferroptosis can promote the secretion of proinflammatory cytokines by activating the NF-κB signaling pathway, and further exacerbating the local inflammatory response. This vicious cycle is a key driver in the progression of RA through multiple mechanisms, including accelerating cartilage and bone destruction, and increased abnormally proliferating synovial cells as well as persistent synovial inflammation (Zhao et al.). Similarly, iron overload can also induce ferroptosis of nucleus pulposus cells and cartilage endplate cells in IVD, and finally contributes to the development of IVDD (Yang et al.). In OA pathogenesis, the central event is the degeneration of articular cartilage, which is maintained by chondrocytes and their ability to balance the synthesis and degradation of extracellular matrix. OA is a multifactorial degenerative joint disease influenced by a combination of genetic, biomechanical, and environmental factors. Key contributors include aging, joint injury, obesity, and abnormal joint loading. These factors elevate ROS production, reduce chondrocyte autophagy, promote chondrocyte apoptosis, and upregulate matrix-degrading enzyme. This perturbs the catabolic and anabolic balance in cartilage, contributing to cartilage degeneration, joint inflammation, and subsequent bone remodeling. These changes hinder the proper transmission of mechanical loads to chondrocyte, amplifying cell death of chondrocyte and ultimately resulting in the occurrence and progression of OA (Li et al.). Recent results from bioinformatic prediction and *in vitro* studies revealed that cuproptosis may also play important roles in RA, IVDD, and OA (Xiang et al.). In addition, many other RCD modes or their cross-talks have been proved to be implicated in the development and progression of above and other musculoskeletal diseases. Despite these insights, significant gaps remain in our understanding of the exact mechanisms and roles of RCD in musculoskeletal diseases. Bridging these gaps is essential for the development of effective prevention and treatment strategies, which will ultimately enhance the outcomes for patients suffering from musculoskeletal diseases.

